# ITGA1 promotes osteogenic differentiation of human periodontal ligament stem cells via FAK-mediated PI3K–Akt activation

**DOI:** 10.1186/s13287-026-05139-6

**Published:** 2026-07-03

**Authors:** Jie Tan, Haoli Tang, Shuangshuang Peng, Xin Li, Meng Dai, Maike Xu, Shijia Zhu, Rui Zhang, Chengcheng Pi, Fuheng Jiang, Kun Yang, Jun Li

**Affiliations:** 1 Department of Implantology, Hospital of Stomatology, Zunyi Medical University, Zunyi, 563000 Guizhou China; 2 Oral Disease Research Key Laboratory of Guizhou Tertiary Institution, School of Life Science, Zunyi Medical University, Zunyi, 563000 Guizhou China; 3 Department of Periodontology, Hospital of Stomatology, Zunyi Medical University, Zunyi, 563000 Guizhou China; 4 Department of Endodontics, Hospital of Stomatology, Zunyi Medical University, Zunyi, 563000 Guizhou China

**Keywords:** Human periodontal ligament stem cells, Osteogenic differentiation, Integrin α1, Focal adhesion kinase, PI3K–Akt signalling pathway

## Abstract

**Background:**

Human periodontal ligament stem cells (hPDLSCs) possess promising potential for bone regeneration; however, the regulation of their osteogenic differentiation remains incompletely understood. Integrin α1 (ITGA1) has been implicated in multiple cellular processes, but its role in hPDLSC osteogenesis requires further investigation.

**Methods:**

ITGA1 was overexpressed in hPDLSCs using an adenoviral vector, and osteogenic differentiation was evaluated by alkaline phosphatase (ALP) staining and gene expression analysis. Loss-of-function experiments were performed using ITGA1 knockdown. Data-independent acquisition (DIA) proteomics, protein–protein interaction (PPI) analysis, immunoprecipitation–mass spectrometry (IP–MS), and co-immunoprecipitation (Co-IP) were employed to explore the underlying mechanisms. Functional validation was performed using gene silencing and pharmacological inhibition, and in vivo efficacy was assessed in a rat mandibular bone defect model.

**Results:**

Overexpression of ITGA1 enhanced osteogenic differentiation, while its knockdown produced the opposite effect. Proteomic analysis identified multiple differentially expressed proteins, including integrin family members, suggesting potential functional specificity. ITGA5 knockdown did not impair ITGA1-mediated osteogenesis. PPI and Co-IP analyses supported an interaction between ITGA1 and focal adhesion kinase (FAK). Inhibition of FAK or PI3K–Akt signalling reduced the pro-osteogenic effects of ITGA1. In vivo, transplantation of ITGA1-overexpressing hPDLSCs promoted bone regeneration in rat mandibular defects.

**Conclusions:**

Activation of the FAK/PI3K–Akt signalling pathway contributes to the promoting effect of ITGA1 on osteogenic differentiation of hPDLSCs. These findings provide insight into integrin-mediated regulation of hPDLSC osteogenesis and may inform future strategies for bone regeneration.

**Supplementary Information:**

The online version contains supplementary material available at https://doi.org/10.1186/s13287-026-05139-6.

## Introduction

Alveolar bone, a crucial tissue in the oral and maxillofacial region, is frequently destroyed by inflammation, trauma, and tumours, leading to complications such as tooth loss, facial collapse, and impaired masticatory function, which complicate prosthetic rehabilitation [1–3]. While grafts utilizing autogenous bone, allografts, xenografts, and synthetic bone substitutes have been shown to be effective at repairing small alveolar defects [4, 5], the reconstruction of critical-sized defects remains challenging because of limitations such as donor site morbidity, limited tissue availability, and graft resorption [6]. Bone tissue engineering, which combines seed cells, bioactive scaffolds, and signalling molecules, offers a promising alternative for achieving alveolar bone regeneration [7, 8].

Human periodontal ligament stem cells (hPDLSCs), a type of mesenchymal stem cell (MSC) of dental origin first identified by Seo et al. in 2004 [9], are easily sourced from extracted teeth and possess a strong self-renewal capacity and multilineage differentiation potential; these cells include osteoblasts, chondrocytes, and cementoblasts [10, 11]. Their low immunogenicity also permits the use of allogeneic hPDLSCs [9, 12]. Consequently, hPDLSCs are widely regarded as excellent cell sources for bone tissue engineering applications [13, 14]. However, the regulatory networks governing the osteogenic differentiation of hPDLSCs and other stem cells have not been fully elucidated. Current bone regeneration techniques often fail to precisely direct the differentiation fate of stem cells in vivo, leading to low osteogenic efficiency and unpredictable outcomes [15, 16]. Identifying key regulatory targets and clarifying their mechanisms is therefore essential for advancing regenerative strategies for alveolar bone.

Integrins are glycosylated, transmembrane adhesion receptors composed of noncovalently linked α and β subunits [17]. A total of 18 α and 8 β integrin subunits have been identified in vertebrates, enabling integrins to form at least 24 distinct heterodimers [17, 18]. Expressed on most cell surfaces, integrins mediate cell–extracellular matrix (ECM) and cell–cell interactions [19, 20]. Ligand binding initiates integrin clustering and the recruitment of adaptor proteins to cytoplasmic domains, activating intracellular signalling pathways that regulate vital cellular processes such as metabolism, survival, proliferation, and differentiation [21–23]. Integrin α1 (ITGA1), a transmembrane receptor mediating cell–extracellular matrix interactions, has been implicated in various biological processes, including cell proliferation, migration, and tumour progression [24, 25]. Previous studies have shown that ITGA1 participates in signalling pathways such as PI3K–Akt and p53 signalling in different cell types [26, 27]. However, its role in regulating osteogenic differentiation of hPDLSCs remains unclear. Our previous work revealed that p75 neurotrophin receptor-positive (p75NTR^+^) hPDLSCs, a cranial neural crest-derived subpopulation, exhibit superior osteogenic potential correlated with high ITGA1 expression. ITGA1 knocked down (KD) inhibited osteogenesis in p75NTR^+^ hPDLSCs, whereas ITGA1 overexpression (OE) increased osteogenesis in p75NTR^−^ hPDLSCs [28]. Based on these observations, we hypothesized that ITGA1 may regulate osteogenic differentiation of hPDLSCs through integrin-associated signalling pathways.

The present study aimed to investigate the role of ITGA1 in hPDLSC osteogenesis and to explore the associated molecular mechanisms. We established an OE-ITGA1 hPDLSC model and performed comparative proteomic and pathway analyses to identify key differentially expressed proteins (DEPs) and related signalling pathways. Our findings provide a basis for enhancing hPDLSC-based bone tissue engineering strategies for alveolar bone regeneration.

## Materials and methods

### Cell culture

Human tissue samples were obtained from the Hospital of Stomatology, Zunyi Medical University, with approval from the Ethics Committee of Zunyi Medical University (Approval No. [2022] 1–037), and informed patient consent was obtained. All procedures complied with the Declaration of Helsinki. Healthy premolars or impacted teeth extracted for orthodontic reasons were collected from systemically healthy donors aged 18–25 years. Periodontal ligament tissues were gently scraped from the middle third of the tooth roots, minced, and centrifuged. The supernatant was discarded, and the tissue pellets were resuspended in α-minimum essential medium (α-MEM; Gibco, USA) supplemented with 10% foetal bovine serum (FBS; OriCell, China) and 1% penicillin–streptomycin–amphotericin (Solarbio, China). Cells were seeded into culture flasks and maintained at 37 °C in a humidified atmosphere containing 5% CO₂. The medium was changed every 3–5 days. Upon reaching 90% confluence, the cells were passaged using 0.25% trypsin (Solarbio) and subcultured at a 1:2 ratio. Cells at passage 3 were used for subsequent experiments. Cells from different donors were used across experiments, and key findings were reproduced in independent biological replicates.

### Flow cytometry

After being harvested, 5 × 10⁵ hPDLSCs were resuspended in phosphate-buffered saline (Biosharp, China) to obtain a single-cell suspension. According to the criteria for defining MSCs established by the International Society for Cellular Therapy [29], the cells were incubated for 15 min at room temperature in the dark with fluorescently labelled antibodies against CD44, CD73, CD90, and CD105 (BioLegend, USA), which are positive markers, and CD34 and CD11b (Absin, China), which are negative markers. Flow cytometric analysis was performed using a flow cytometer (Challenbio, China) to determine surface marker expression.

### Osteogenic induction, alkaline phosphatase (ALP) staining, and alizarin red S staining

hPDLSCs (2 × 10⁵) were seeded in 6-well plates and cultured in osteogenic induction medium consisting of α-MEM supplemented with 5% FBS, 1% penicillin–streptomycin–amphotericin, 10 µmol/L dexamethasone (Solarbio), 50 mg/L ascorbic acid (Solarbio), and 10 mmol/L β-glycerophosphate (Solarbio). The medium was replaced every 3 days. Early osteogenic differentiation and late mineralization were assessed by specific staining after 5 and 28 days of induction, respectively. After 5 days, ALP activity was evaluated using a staining kit (Beyotime, China) following fixation with 4% paraformaldehyde (Solarbio); the staining intensity was quantified from micrographs of three independent experiments using ImageJ software. After 28 days, mineralized nodule formation was visualized by Alizarin Red S staining (OriCell) postfixation, and representative images were captured under a microscope (MF53-N, Mshot, China).

### Adipogenic induction and oil red O staining

Adipogenic differentiation was performed using a commercial human mesenchymal stem cell adipogenic differentiation kit (OriCell) according to the manufacturer’s instructions. Briefly, the reagents were diluted in α-MEM containing 5% FBS and 1% penicillin–streptomycin–amphotericin B to prepare adipogenic induction media A and B. Confluent hPDLSCs (2 × 10⁵) were cultured in medium A for 3 days, followed by medium B for 1 day, and this 4-day cycle was repeated for 28 days. After induction, the cells were fixed with 4% paraformaldehyde and stained with freshly prepared Oil Red O working solution (OriCell; stock solution: water = 3:2). Lipid droplets were observed and photographed under a microscope.

### Chondrogenic induction and alcian blue staining

Chondrogenic differentiation was conducted using an OriCell commercial kit supplemented with α-MEM containing 5% FBS and 1% penicillin–streptomycin–amphotericin B. hPDLSCs (4 × 10⁵) were washed, pelleted in 15 mL tubes, and cultured upright in chondrogenic induction medium for 21 days, with the medium changed every 3 days. The cell pellets were fixed with 4% paraformaldehyde, embedded in paraffin, sectioned, and stained with Alcian blue (OriCell) before microscopic observation.

### Adenoviral transduction

The OE-ITGA1 construct was delivered using a CMV promoter-driven adenoviral vector that does not integrate into the host genome. ITGA1 primer sequences (Sangon, China) are listed in Table S1. The multiplicity of infection (MOI) was optimised based on preliminary experiments (Supplementary Figure S1A–C). hPDLSCs (2 × 10⁵) were plated in 6-well plates and then transduced with either OE-ITGA1 adenovirus or an empty negative control (NC) adenoviral vector lacking the target gene sequence (MOI = 10; Sangon). Both vectors were constructed using the same adenoviral backbone and applied under identical experimental conditions. Transduction was performed using polybrene (5 µg/mL; Hanbio, China) for 4 h.

### Quantitative real-time polymerase chain reaction (qRT-PCR)

Total RNA was isolated using a commercial extraction kit (Solarbio) and quantified with a spectrophotometer (Thermo Scientific, USA). Total RNA was treated with DNase I to eliminate genomic DNA contamination prior to reverse transcription. RNA was reverse-transcribed into cDNA using PrimeScript™ RT Master Mix (Takara, Japan) on a T100 Thermal Cycler (Bio-Rad, USA). qRT-PCR was carried out using TB Green™ Premix Ex Taq™ II (Takara) in a 10 µL reaction on a CFX96 Real-Time System (Bio-Rad). GAPDH was used as an internal control, and the relative gene expression data from three independent biological replicates were calculated using the 2^⁻ΔΔCT^ method. The stability of GAPDH expression under experimental conditions was preliminarily validated. The primer sequences (Sangon) are listed in Table S2.

### Transfection of small interfering RNA (siRNA)

hPDLSCs (2 × 10^5^) were seeded in 6-well plates and then transfected with ITGA1, ITGA5, FAK, or NC siRNA (100 pmol/well; Sangon) using Lipofectamine 2000 (1:400; Invitrogen, USA) for 6 h. The primer sequences (Sangon) are listed in Table S3.

### Western blot analysis

Cell lysates were prepared using RIPA buffer (Epizyme, China) containing protease and phosphatase inhibitors (Solarbio). Protein concentrations were determined using a BCA assay kit (Solarbio). Equal amounts of protein (20 µg/lane) were separated by SDS–PAGE and transferred to PVDF membranes (Merck-Millipore, Germany). The membranes were blocked with 5% bovine serum albumin in TBST for 1 h at room temperature and incubated overnight at 4 °C with the following primary antibodies: rabbit anti-human ITGA1, COL1A1, RUNX2, ALP (all 1:1000; Abcam, UK), ITGA5 (1:2000; Epizyme), FAK, phosphor (p)-FAK (Tyr397), PI3K p110α, p-PI3K p85 (Tyr458)/p55 (Tyr199), Akt, p-Akt (1:1000–1:2000; Cell Signaling Technology, USA), and GAPDH (1:30000; Abcam). After being washed, the membranes were incubated with a horseradish peroxidase–conjugated goat anti-rabbit IgG secondary antibody (1:5000; Epizyme) for 1 h at room temperature. Bands from three independent experiments were visualized using ECL chemiluminescence and quantified with ImageJ.

### Data-independent acquisition (DIA) quantitative proteomics analysis

DIA-based quantitative proteomics was performed by Novogene Co., Ltd. (Beijing, China). Cells subjected to 5 days of osteogenic induction, specifically OE-ITGA1 and NC hPDLSCs, were collected, with three independent biological replicates per group. Cell samples were lysed in a buffer containing 8 mol/L urea and 0.1 mol/L TEAB (pH 8.5), followed by in-solution tryptic digestion. The resulting peptides were analysed on an Orbitrap Astral mass spectrometer (Thermo Scientific) to acquire raw data. The raw data were then searched against the UniProt database to generate the proteomic dataset. Differential expression analysis was performed using Student’s t test, with proteins satisfying the criteria of a P value < 0.05 and a fold change (FC) > 1.25 (upregulated) or < 0.80 (downregulated) defined as DEPs.

### Protein–protein interaction (PPI) analysis

DEPs identified by DIA-based quantitative proteomics were analysed using the Search Tool for the Retrieval of Interacting Genes (STRING; https://string-db.org, version 12.0) [30]. A minimum required interaction score of 700 was set to screen for proteins with potential functional or physical interactions with ITGA1.

### Immunoprecipitation–mass spectrometry (IP–MS) and coimmunoprecipitation (Co-IP)

OE-ITGA1 and NC hPDLSCs subjected to 5 days of osteogenic induction were collected and lysed to obtain whole-cell extracts. The lysates were incubated overnight at 4 °C with a mouse anti-human ITGA1 (1:100; Santa Cruz Biotechnology, USA), a rabbit anti-human FAK (1:100; Cell Signaling Technology), a mouse (G3A1) mAb IgG1 isotype control (1:50; Cell Signaling Technology), or a rabbit IgG control (1:50; Proteintech, China), followed by incubation with protein A/G magnetic beads (MedChemExpress, USA) for 3 h at 4 °C. After washing, the bound proteins were eluted using SDS–PAGE loading buffer (Epizyme), and the supernatant was collected by centrifugation. For IP–MS, the immunoprecipitated samples were subjected to qualitative mass spectrometry using a Q Exactive HF mass spectrometer (Thermo Scientific) under standard data-dependent acquisition conditions, and the resulting data were searched against the UniProt Knowledgebase for protein identification. For Co-IP, the eluted proteins were analysed by Western blot.

### Inhibition and activation of the PI3K signalling pathway

The PI3K inhibitor LY294002 or agonist 740 Y-P (both 10 µmol/L; MedChemExpress) was added directly to the osteogenic induction medium during hPDLSC culture.

### Establishment of the rat mandible defect model

All animal experiments were approved by the Animal Welfare and Ethics Committee of Zunyi Medical University (Approval No. ZMU21-2203-520). The work has been reported in line with the ARRIVE guidelines 2.0. A total of thirty healthy male Sprague–Dawley (SD) rats (6–8 weeks, 220–250 g) were randomly allocated into five experimental groups (*n* = 6) using a computer-generated randomization sequence. The sample size was determined based on previous studies employing similar critical-size defect models to ensure adequate statistical power [31]. Animals were group-housed under specific pathogen-free conditions in a controlled environment (12-hour light/dark cycle, 22 ± 1 °C, 50 ± 10% humidity) with ad libitum access to standard rodent diet and water. A 5 wt% GelMA (LinGel, China) hydrogel containing 0.2 wt% photoinitiator LAP (LinGel) was sterilized by 0.22 μm filtration (Merck-Millipore) and mixed with 5-day osteogenically induced hPDLSCs from different groups at 5 × 10⁶ cells/mL. The cellular groups were: NC hPDLSCs (control), and OE-ITGA1, OE-ITGA1 + LY294002, OE-ITGA1 + KD-FAK, and OE-ITGA1 + KD-FAK + 740 Y-P hPDLSCs (experimental groups). Under anesthesia induced and maintained with 3% isoflurane (RWD, China), a cylindrical critical-size defect (4 mm diameter, 2 mm depth) was created on the lateral aspect of the right mandible, 4 mm below the third molar. The defect was filled with 100 µL of the respective cell-laden hydrogel and crosslinked under blue light for 15 s before wound closure. Postoperatively, animals received analgesia and were monitored daily for signs of distress or infection. The rats were maintained under the standard conditions described above for 28 days. At the endpoint, rats were deeply anesthetized by continuous inhalation of isoflurane (RWD, China) until loss of toe-pinch reflex, followed by euthanasia via intraperitoneal injection of a lethal dose of tribromoethanol (385 mg/kg; HTQ biology, China). Death was confirmed by the absence of respiration and heartbeat. Mandibles were harvested from all animals for subsequent analysis. No animals were excluded from the analysis. The surgeon procedure and outcome assessment was performed in a blinded manner.

### Micro-CT analysis

Mandibles from rats were fixed in 4% paraformaldehyde at 4 °C for 48 h and then scanned using a micro-CT scanner (Bruker, Germany). The X-ray source was set at 50 kV and 72 µA with a resolution of 6 μm. Three-dimensional reconstruction was performed using NRecon software, and subsequent analysis of the bone defect region was conducted with CT analyser software to quantify bone morphometric parameters: bone volume/total volume (BV/TV), trabecular number (Tb.N), trabecular thickness (Tb.Th), and trabecular separation (Tb.Sp).

### Haematoxylin and eosin (H&E) staining

Mandibular bone samples were fixed in 4% paraformaldehyde and decalcified in EDTA (Solarbio). Following dehydration, the samples were embedded in paraffin and cut into 3 μm-thick slices using a microtome (Leica Biosystems, Germany). The sections were then stained with H&E (Solarbio), dehydrated, mounted, and finally examined under a microscope.

### Masson staining

Mandibular bone samples were embedded, sectioned, and sequentially stained with potassium chromate (Paini Technology, China), Ponceau S-Fuchsin (Aladdin, USA), phosphomolybdic acid (Solarbio), and aniline blue (Aladdin). Finally, the stained sections were dehydrated, mounted, and observed under a microscope.

### Immunofluorescence staining

Following deparaffinization, mandibular tissue sections were blocked with 5% goat serum (Solarbio) at room temperature for 30 min. The sections were subsequently incubated overnight at 4 °C in a humidified chamber with the following primary antibodies: mouse anti-rat ITGA1 (1:200; Santa Cruz Biotechnology) and rabbit anti-rat RUNX2 (1:100; Abcam). After being washed, the sections were incubated with secondary antibodies—Cy™3-conjugated AffiniPure™ Goat Anti-Mouse IgG (H + L) and Alexa Fluor^®^ 488-conjugated AffiniPure™ Goat Anti-Rabbit IgG (H + L) (both 1:400; Jackson ImmunoResearch, USA)—for 1 h at room temperature in the dark. The cell nuclei were counterstained with DAPI (Biosharp) for 5 min under light-protected conditions. Finally, the sections were mounted with anti-fade mounting medium (Solarbio) and observed under a fluorescence microscope. Images were captured, and fluorescence intensity was quantified using ImageJ.

### Statistical analysis

All the quantitative data are presented as the mean ± standard deviation (SD). All experiments were performed with at least three independent biological replicates (*n* ≥ 3), and “n” represents biological replicates. Statistical analyses were performed using GraphPad Prism 10.0 software. Comparisons between two groups were analysed by an unpaired two-tailed Student’s t test. Comparisons among multiple groups were analysed by one-way analysis of variance (one-way ANOVA) with Tukey’s post hoc test. A P value < 0.05 was considered to indicate statistical significance.

## Results

### OE-ITGA1 promotes osteogenic differentiation of hPDLSCs

**Fig. 1:**
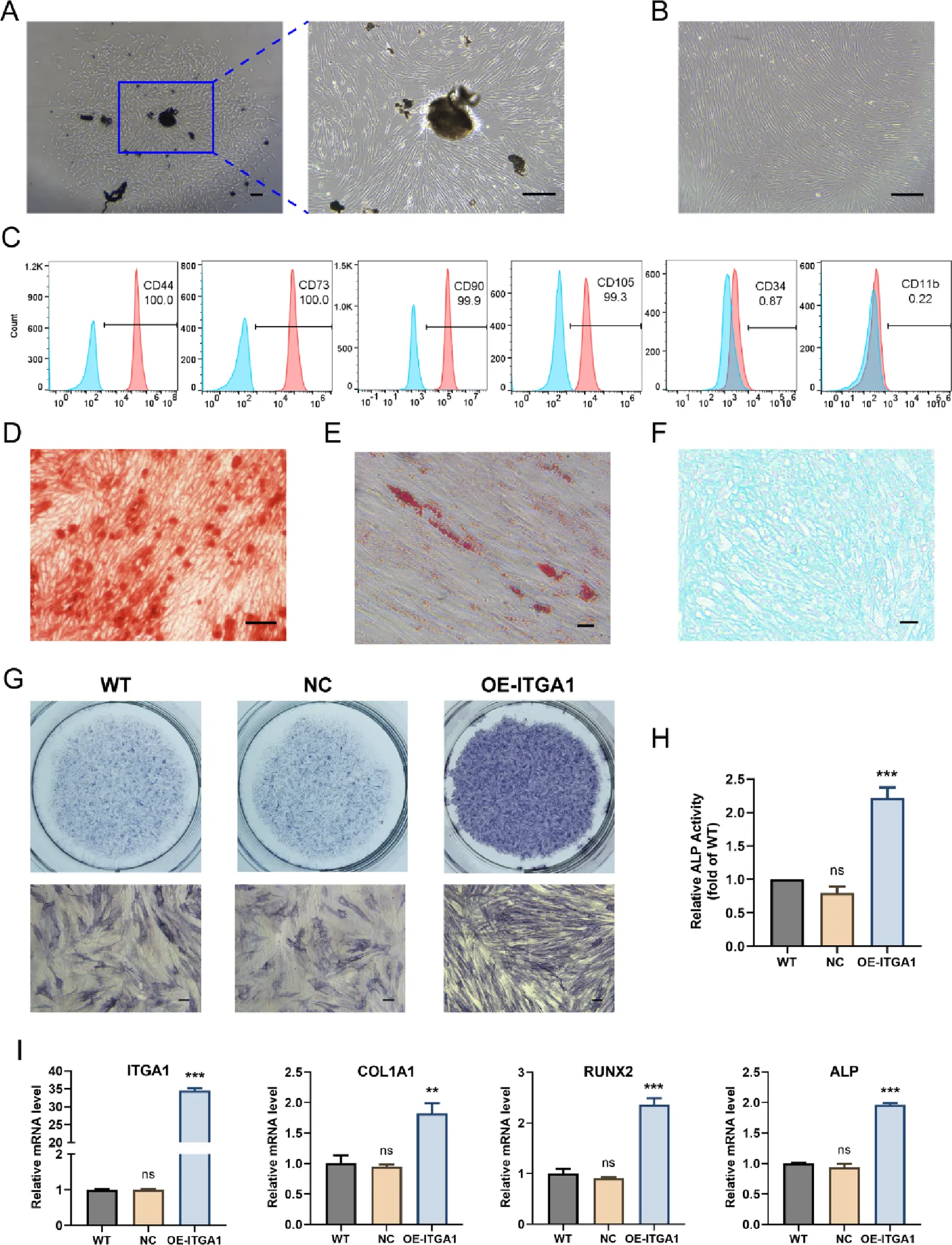
Regulatory role of ITGA1 in osteogenic differentiation of hPDLSCs. **A**–**F** Isolation and characterization of hPDLSCs. **A** Representative image of primary hPDLSCs. Scale bar = 200 μm. **B** Morphology of passage 3 hPDLSCs. Scale bar = 200 μm. **C** Flow cytometric analysis of hPDLSC surface markers. **D **Alizarin Red S staining following 28 days of osteogenic induction. Scale bar = 200 μm. **E** Oil Red O staining after 28 days of adipogenic induction. Scale bar = 20 μm. **F** Alcian Blue staining following 21 days of chondrogenic induction. Scale bar = 50 μm. **G**–**I**. OE-ITGA1 promotes osteogenic differentiation of hPDLSCs. **G** ALP staining after 5 days of osteogenic induction. Scale bar = 100 μm. **H** Semi-quantitative analysis of ALP staining intensity measured by ImageJ (*n* = 3). **I** Relative mRNA expression levels of *ITGA1*, *COL1A1*, *RUNX2*, and *ALP* measured by qRT-PCR after 5 days of osteogenic induction. *GAPDH* was used as the internal control (*n* = 3). Data are presented as mean ± SD. Statistical significance: *P < 0.05, **P < 0.01, ***P < 0.001

**Fig. 2:**
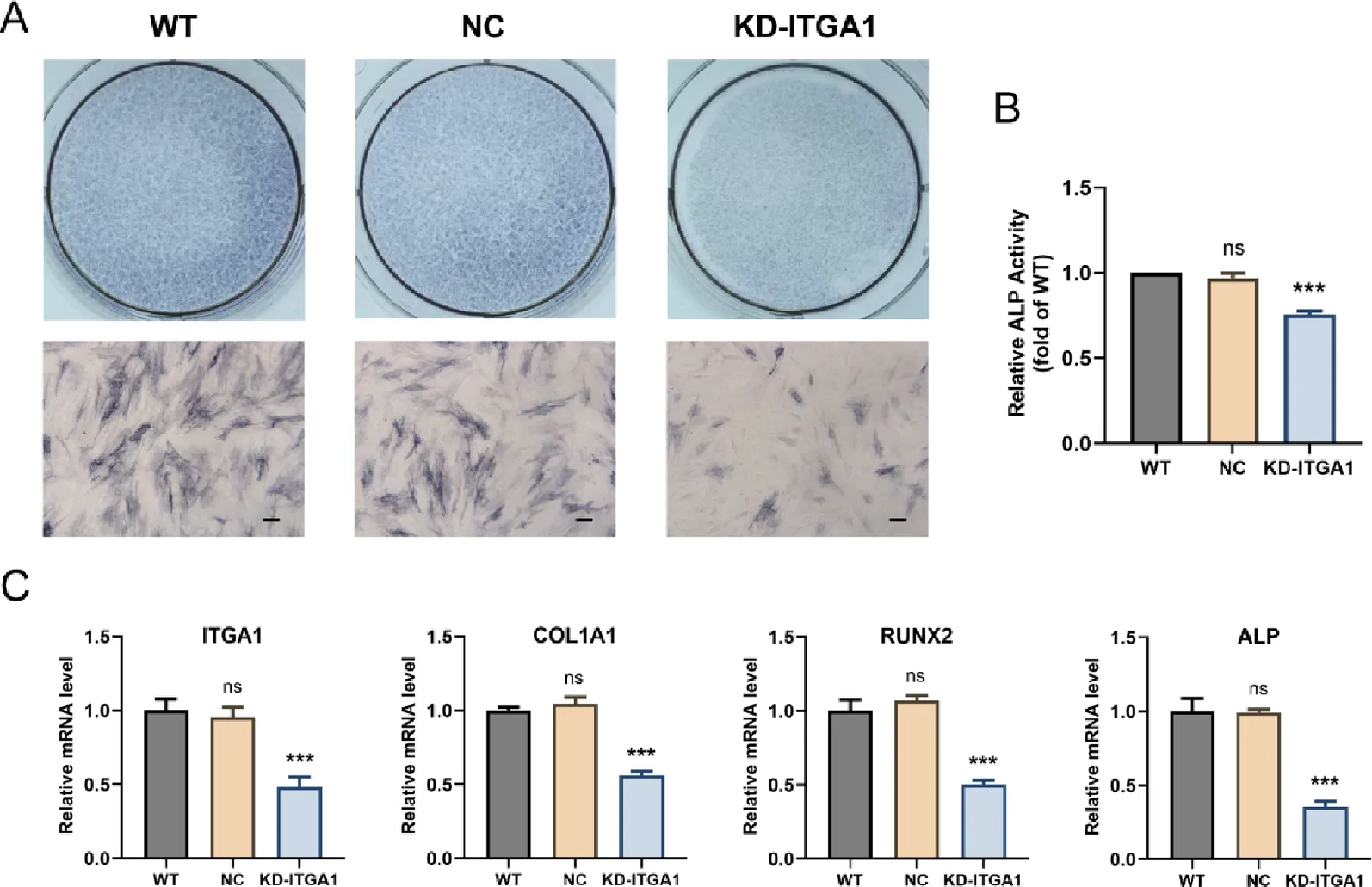
Effects of KD-ITGA1 on osteogenic differentiation of hPDLSCs. **A** ALP staining of hPDLSCs following KD-ITGA1 after 5 days of osteogenic induction. Scale bar = 100 μm. **B** Semi-quantitative analysis of ALP staining intensity using ImageJ (*n* = 3). **C** Relative mRNA expression levels of *ITGA1*, *COL1A1*, *RUNX2*, and *ALP* measured by qRT-PCR after 5 days of osteogenic induction. *GAPDH* was used as the internal control (*n* = 3). Data are presented as mean ± SD. Statistical significance: *P < 0.05, **P < 0.01, ***P < 0.001

Primary cultured hPDLSCs displayed a homogeneous, spindle-shaped morphology with an orderly arrangement (Fig. 1A–B). Flow cytometry analysis confirmed the identity of the mesenchymal stem cells, revealing high expression of positive markers (CD44, CD73, CD90, and CD105) and low expression of negative markers (CD34 and CD11b) (Fig. 1C). Following osteogenic induction, the cells formed distinct mineralized nodules, as visualized by Alizarin Red S staining (Fig. 1D), confirming their osteogenic potential. Adipogenic induction led to the appearance of cytoplasmic lipid droplets, as detected by Oil Red O staining (Fig. 1E), indicating adipogenic capacity. Chondrogenic induction produced Alcian blue-positive extracellular matrix (Fig. 1F), demonstrating chondrogenic potential through acidic proteoglycan deposition. To elucidate the role of ITGA1 in hPDLSC osteogenesis, OE-ITGA1 hPDLSCs were generated via adenovirus transduction. Compared with both NC and wild-type (WT) hPDLSCs, OE-ITGA1 hPDLSCs exhibited stronger ALP staining intensity (Fig. 1G–H) and elevated mRNA levels of *ITGA1*, *COL1A1*, *RUNX2*, and *ALP* (Fig. 1I). These findings indicate that OE-ITGA1 enhances the osteogenic differentiation potential of hPDLSCs. In addition to overexpression experiments, KD-ITGA1 significantly reduced osteogenic differentiation, as indicated by decreased ALP activity (Fig. 2A–B) and reduced expression of osteogenic markers (Fig. 2C).

### PI3K–Akt pathway activity is upregulated in OE-ITGA1 hPDLSCs

**Fig. 3:**
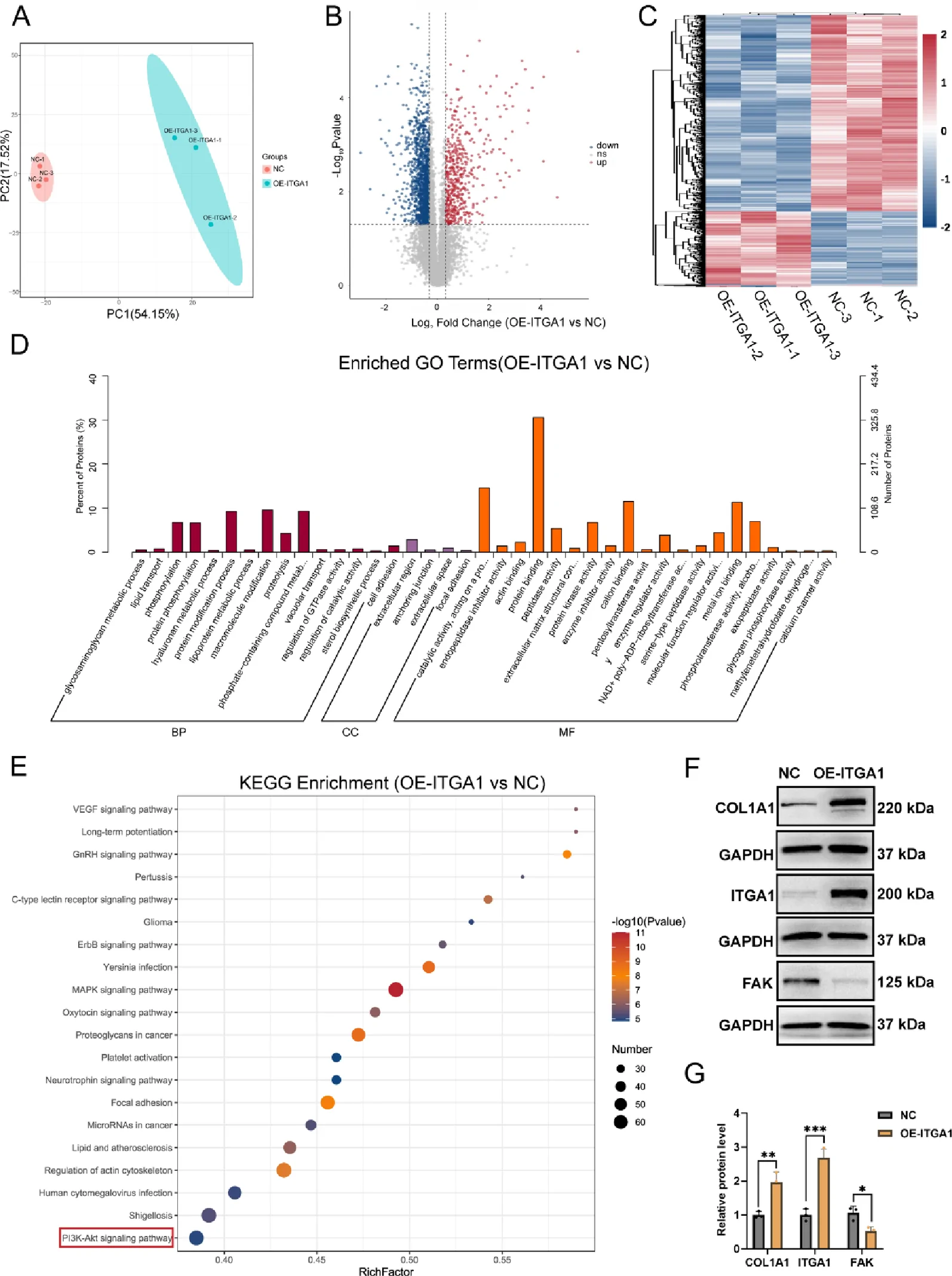
Differential expression protein analysis in OE-ITGA1 hPDLSCs. **A**–**E** DIA-based quantitative proteomic analysis of OE-ITGA1 and NC hPDLSCs after 5 days of osteogenic induction (*n* = 3). **A** Principal component analysis plot. **B** Volcano plot of DEPs. **C** Hierarchical clustering heatmap of DEPs. **D** GO enrichment analysis of DEPs. **E** KEGG pathway enrichment analysis of DEPs. **F** Western blot analysis validating the expression levels of COL1A1, ITGA1, and FAK in OE-ITGA1 hPDLSCs after 5 days of osteogenic induction (Full-length blots are presented in Supplementary Fig. S2). **G** Semi-quantitative analysis of the Western blot results using ImageJ (*n* = 3). Data are presented as mean ± SD. Statistical significance: *P < 0.05, **P < 0.01, ***P < 0.001

**Fig. 4:**
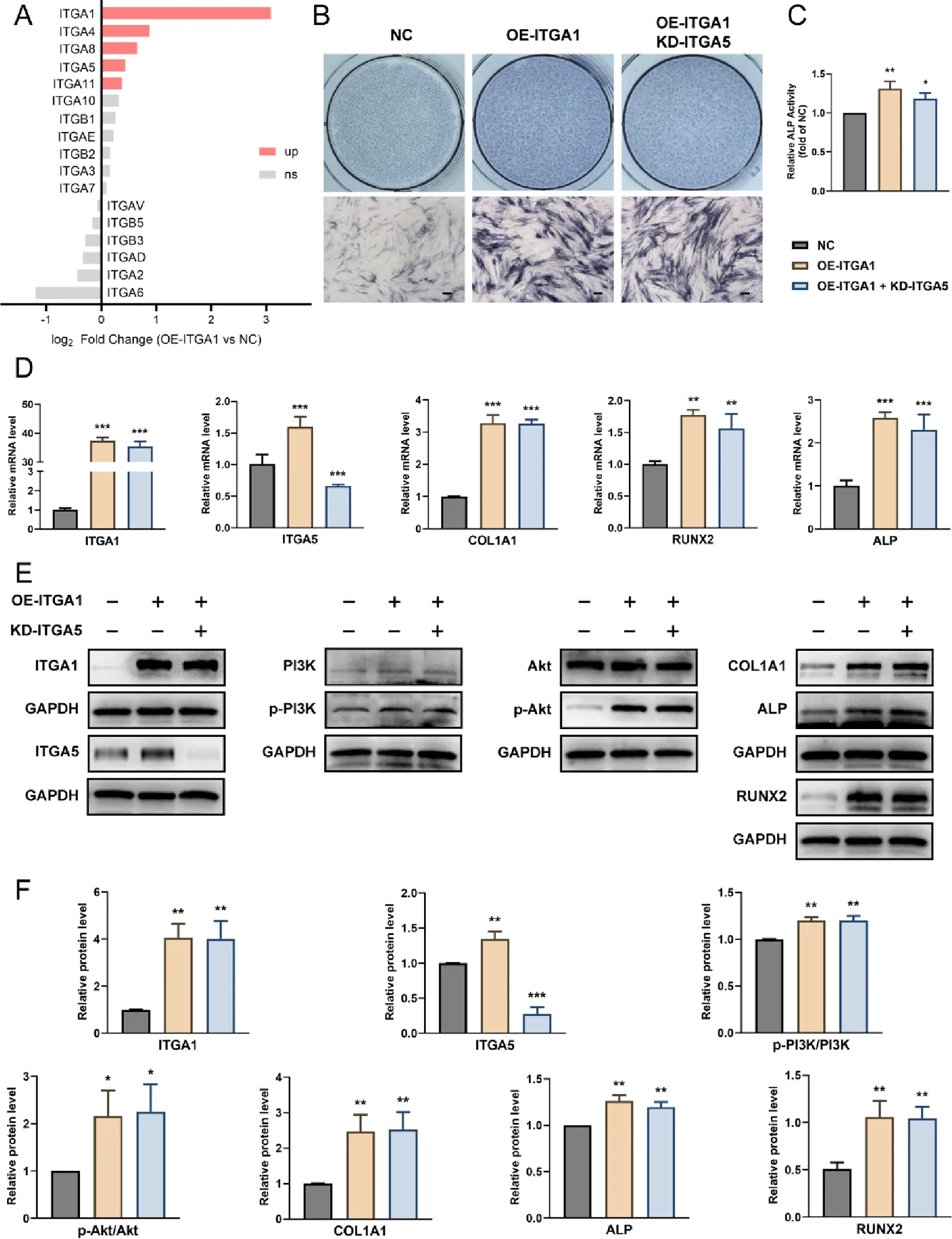
KD-ITGA5 does not impair ITGA1-mediated osteogenic differentiation of hPDLSCs. **A** Expression of integrin family members identified by proteomic analysis in OE-ITGA1 and NC groups. **B** ALP staining of hPDLSCs after 5 days of osteogenic induction. Scale bar = 100 μm. **C** Semi-quantitative analysis of ALP staining intensity using ImageJ (*n* = 3). **D** Relative mRNA expression levels of *ITGA1*, *ITGA5*, *COL1A1*, *RUNX2*, and *ALP* measured by qRT-PCR in hPDLSCs after 5 days of osteogenic induction. *GAPDH* was used as an internal control (*n* = 3). **E** Western blot analysis of ITGA1, ITGA5, PI3K, p-PI3K, Akt, p-Akt, COL1A1, ALP, and RUNX2 after 5 days of osteogenic induction (Full-length blots are presented in Supplementary Fig. S3). **F** Semi-quantitative analysis of protein expression levels using ImageJ (*n* = 3). Data are presented as mean ± SD. Statistical significance: *P < 0.05, **P < 0.01, ***P < 0.001

After osteogenic induction, DIA-based quantitative proteomic analysis revealed a distinct difference in global protein expression between OE-ITGA1 and NC hPDLSCs by principal component analysis (Fig. 3A). Comparative analysis revealed 1,783 DEPs, with 486 upregulated and 1,297 downregulated in OE-ITGA1 cells (Fig. 3B–C) (the complete dataset is provided in Supplementary Table S4). Among these, integrin family members exhibited distinct expression patterns (Fig. 4A). Gene Ontology (GO) analysis revealed significant enrichment of these DEPs in the molecular function category “protein binding” (Fig. 3D). Furthermore, Kyoto Encyclopedia of Genes and Genomes (KEGG) pathway analysis indicated the significant involvement of these genes in the PI3K–Akt signalling pathway (Fig. 3E). Subsequent Western blot validation confirmed that the expression trends of key proteins were consistent with the proteomic findings: compared with those in the control group, the expression levels of ITGA1 and COL1A1 were increased, while the expression level of FAK was decreased in the overexpression group (Fig. 3F–G). Collectively, these findings suggest that OE-ITGA1 enhances osteogenic differentiation in hPDLSCs, likely through activation of the PI3K–Akt pathway. Given the upregulation of ITGA5 in OE-ITGA1 hPDLSCs, its potential involvement was further investigated by siRNA‑mediated silencing. However, KD-ITGA5 did not abolish ITGA1‑mediated osteogenesis (Fig. 4B–D) or activation of the PI3K–Akt signalling pathway (Fig. 4E–F).

### ITGA1 interacts with FAK in hPDLSCs

**Fig. 5:**
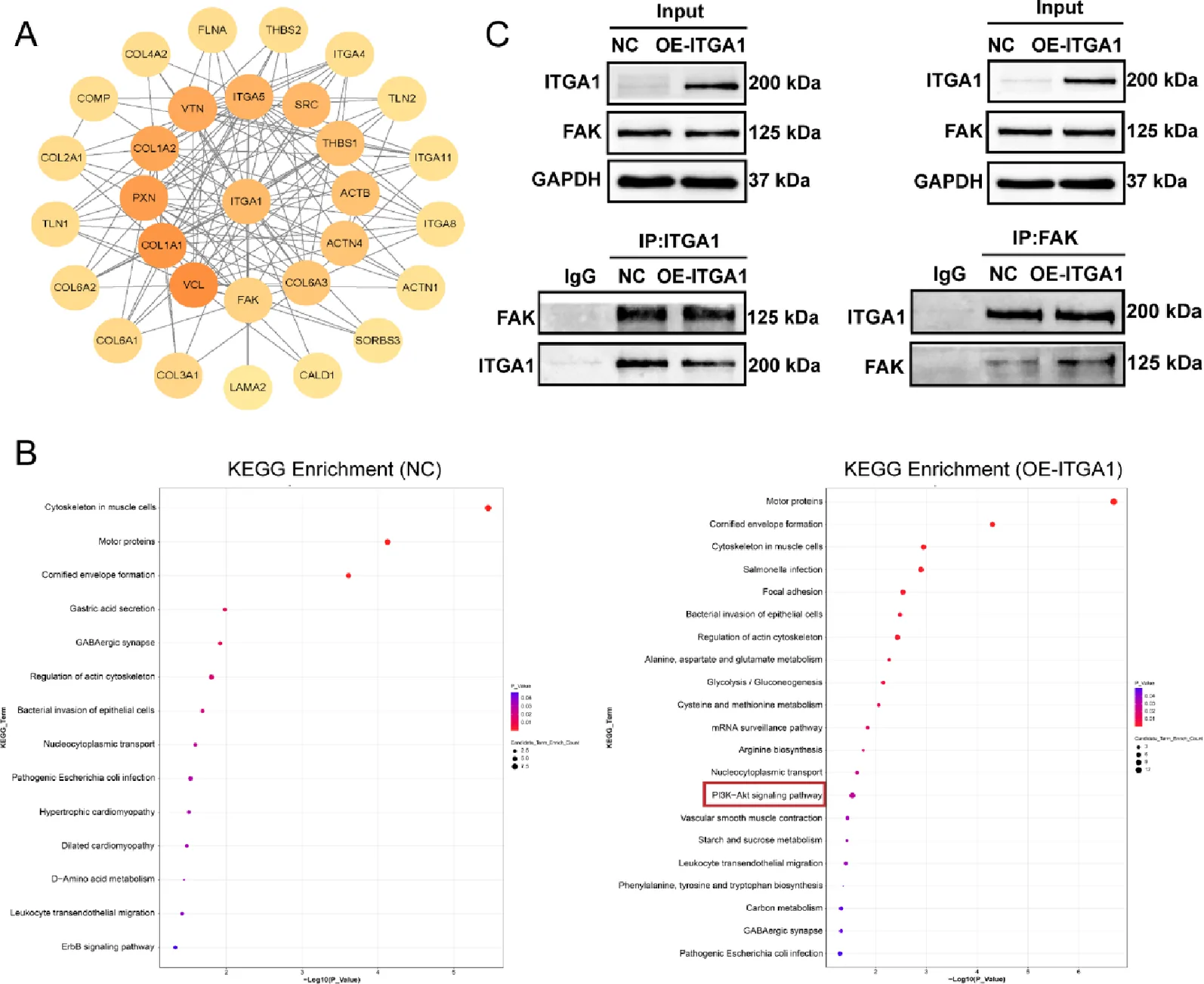
Identification and validation of ITGA1-interacting proteins in hPDLSCs. **A** PPI network analysis identifying candidate proteins associated with ITGA1. **B** KEGG pathway enrichment analysis of proteins identified by IP–MS in OE-IP and NC-IP groups. Pathways are ranked based on protein counts, and significantly enriched pathways are shown. **C** Co-IP assay validating the interaction between ITGA1 and FAK in hPDLSCs after 5 days of osteogenic induction. Input and IgG serve as positive and negative controls, respectively (Full-length blots are presented in Supplementary Fig. S4)

Following osteogenic induction and proteomic analysis, PPI analysis revealed that among the DEPs in OE-ITGA1-hPDLSCs versus NC-hPDLSCs, 29 exhibited an interaction score greater than 700 with ITGA1, with FAK being one of them (Fig. 5A). IP–MS analysis identified multiple ITGA1-associated proteins, suggesting the formation of a protein complex. KEGG enrichment analysis indicated that these proteins were involved in signalling pathways related to osteogenesis (Fig. 5B). Further Co-IP assays confirmed the direct physical interaction between ITGA1 and FAK in both OE-ITGA1 and NC hPDLSCs (Fig. 5C). These data indicate that ITGA1 physically interacts with FAK and may regulate osteogenic differentiation through the FAK-mediated activation of PI3K–Akt signalling.

### ITGA1/FAK/PI3K–Akt signalling positively regulates hPDLSC osteogenesis in vitro

**Fig. 6:**
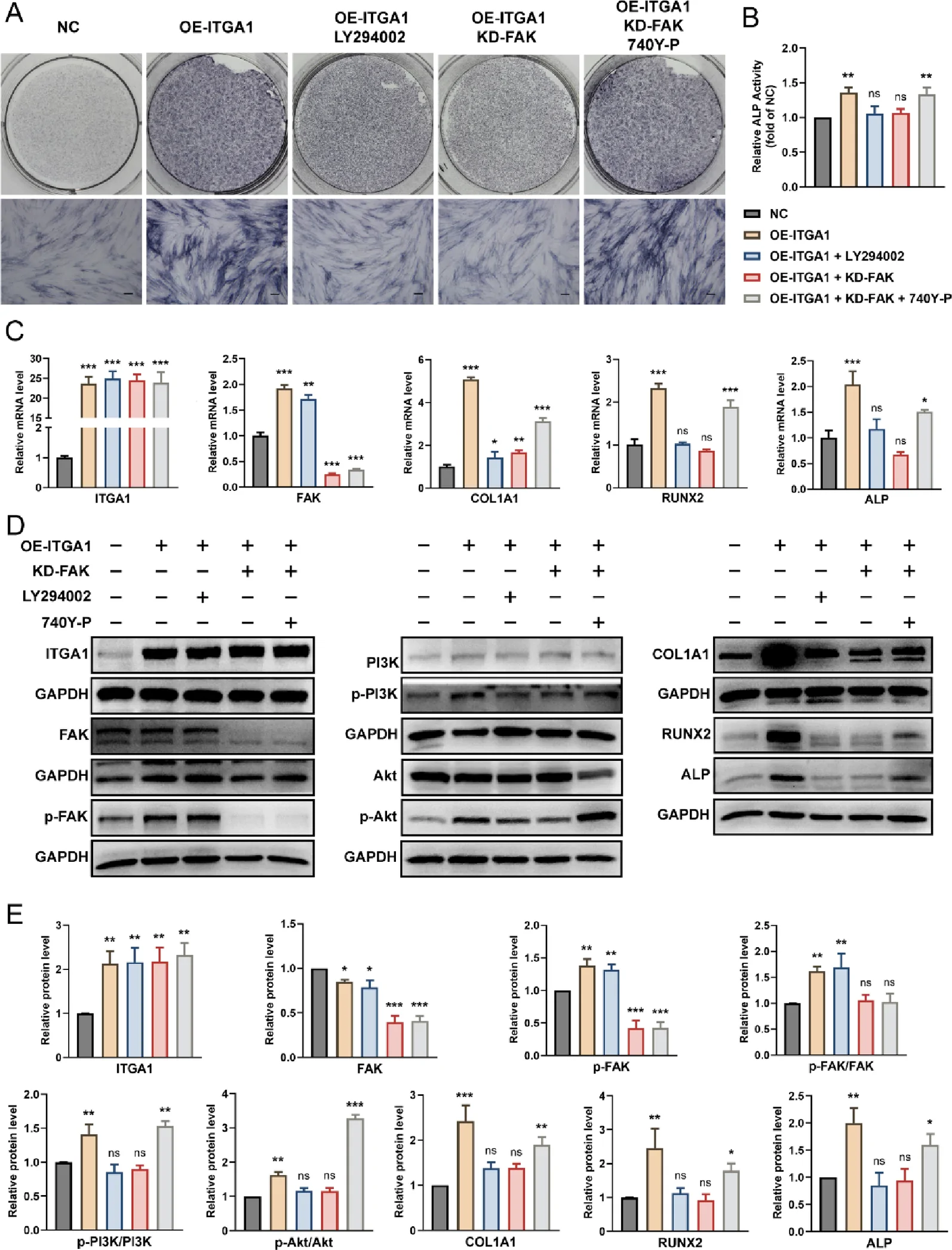
The function of the ITGA1/FAK/PI3K–Akt signalling in osteogenesis of hPDLSCs. **A** ALP staining images of hPDLSCs after 5 days of osteogenic induction. Scale bar = 100 μm. **B** Semi-quantitative analysis of ALP staining intensity performed using ImageJ (*n* = 3). **C** Relative mRNA expression levels of *ITGA1*, *FAK*, *COL1A1*, *RUNX2*, and *ALP* measured by qRT-PCR in hPDLSCs after 5 days of osteogenic induction. *GAPDH* was used as an internal control (*n* = 3). **D** Protein expression levels of ITGA1, FAK, p-FAK, PI3K, p-PI3K, Akt, p-Akt, COL1A1, RUNX2, and ALP were detected by Western blot analysis after 5 days of osteogenic induction (Full-length blots are presented in Supplementary Fig. S5). **E** Semi-quantitative analysis of the Western blot results using ImageJ (*n* = 3). Data are presented as mean ± SD. Statistical significance: *P < 0.05, **P < 0.01, ***P < 0.001

To investigate the role of the ITGA1/FAK/PI3K–Akt signalling pathway in the osteogenic differentiation of hPDLSCs, we silenced FAK using siRNA and further modulated PI3K activity during osteogenic induction with the inhibitor LY294002 or the agonist 740 Y-P. The results showed that both OE-ITGA1 hPDLSCs and OE-ITGA1 + KD-FAK + 740 Y-P hPDLSCs exhibited stronger ALP staining intensity (Fig. 6A–B), higher mRNA (Fig. 6C) and protein (Fig. 6D–E) expression levels of COL1A1, RUNX2, ALP, and increased ratios of p-PI3K/PI3K and p-Akt/Akt compared to the NC group, OE-ITGA1 + LY294002 group, and OE-ITGA1 + KD-FAK group. These findings demonstrate that ITGA1 promotes osteogenic differentiation in hPDLSCs via FAK-dependent activation of the PI3K–Akt signalling pathway. Moreover, pharmacological activation of PI3K can partially rescue the inhibitory effects of KD-FAK, underscoring the central role of this pathway in ITGA1-driven osteogenesis.

### ITGA1/FAK/PI3K–Akt axis facilitates osteogenic differentiation of hPDLSCs in vivo

**Fig. 7:**
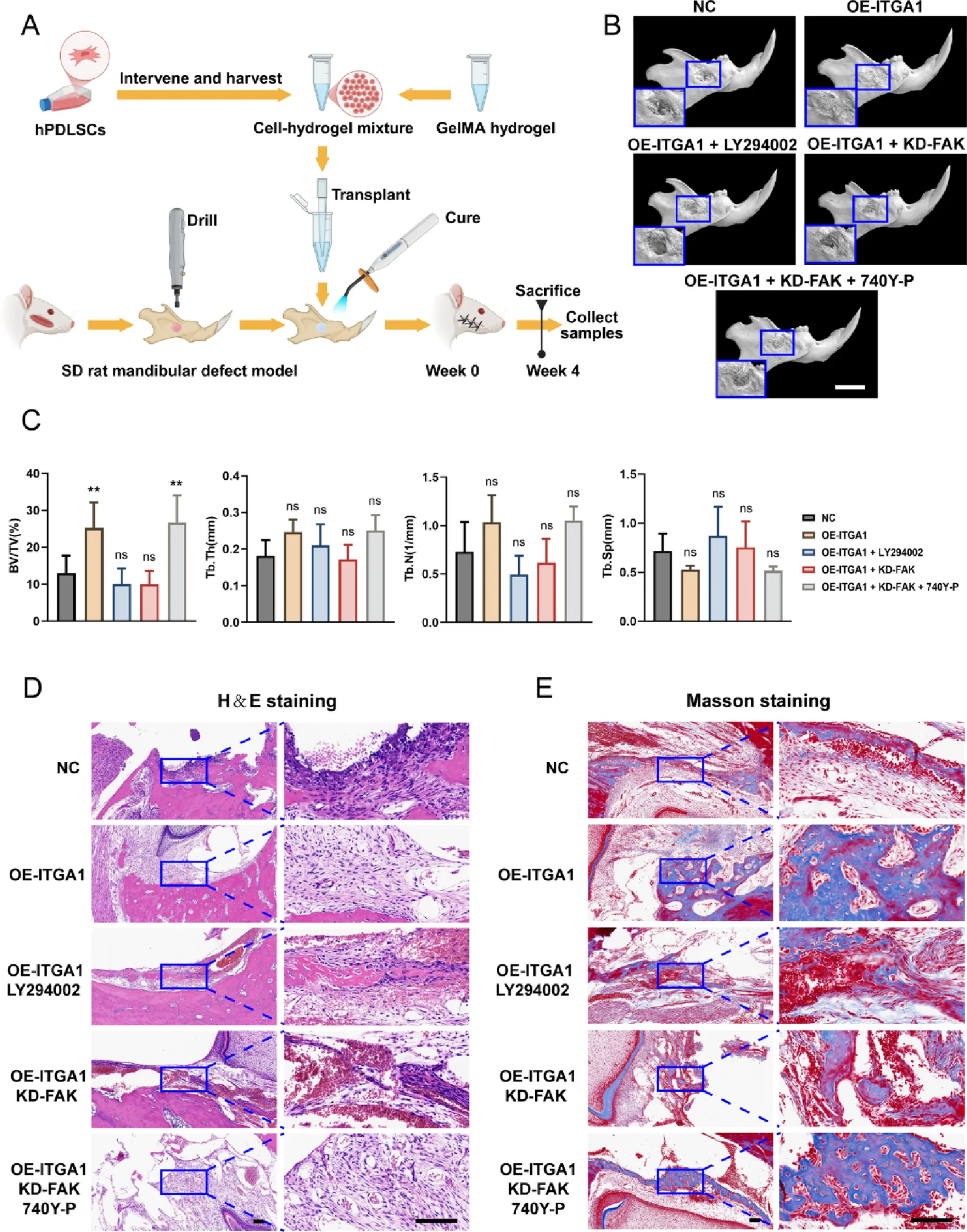
In vivo validation of ITGA1 promoting osteogenic differentiation of hPDLSCs. **A** Schematic illustration of the mandibular bone defect repair in SD rats using hPDLSCs. The figure was created with BioRender.com. **B** Representative micro-CT images of the mandibular bone defect areas. Scale bar = 5 mm. **C** Quantitative analysis of the micro-CT results (*n* = 6). **D** H&E staining of the mandibular defect areas. Scale bar = 100 μm. **E** Masson staining of the mandibular defect areas. Scale bar = 100 μm. Data are presented as mean ± SD. Statistical significance: *P < 0.05, **P < 0.01, ***P < 0.001

**Fig. 8:**
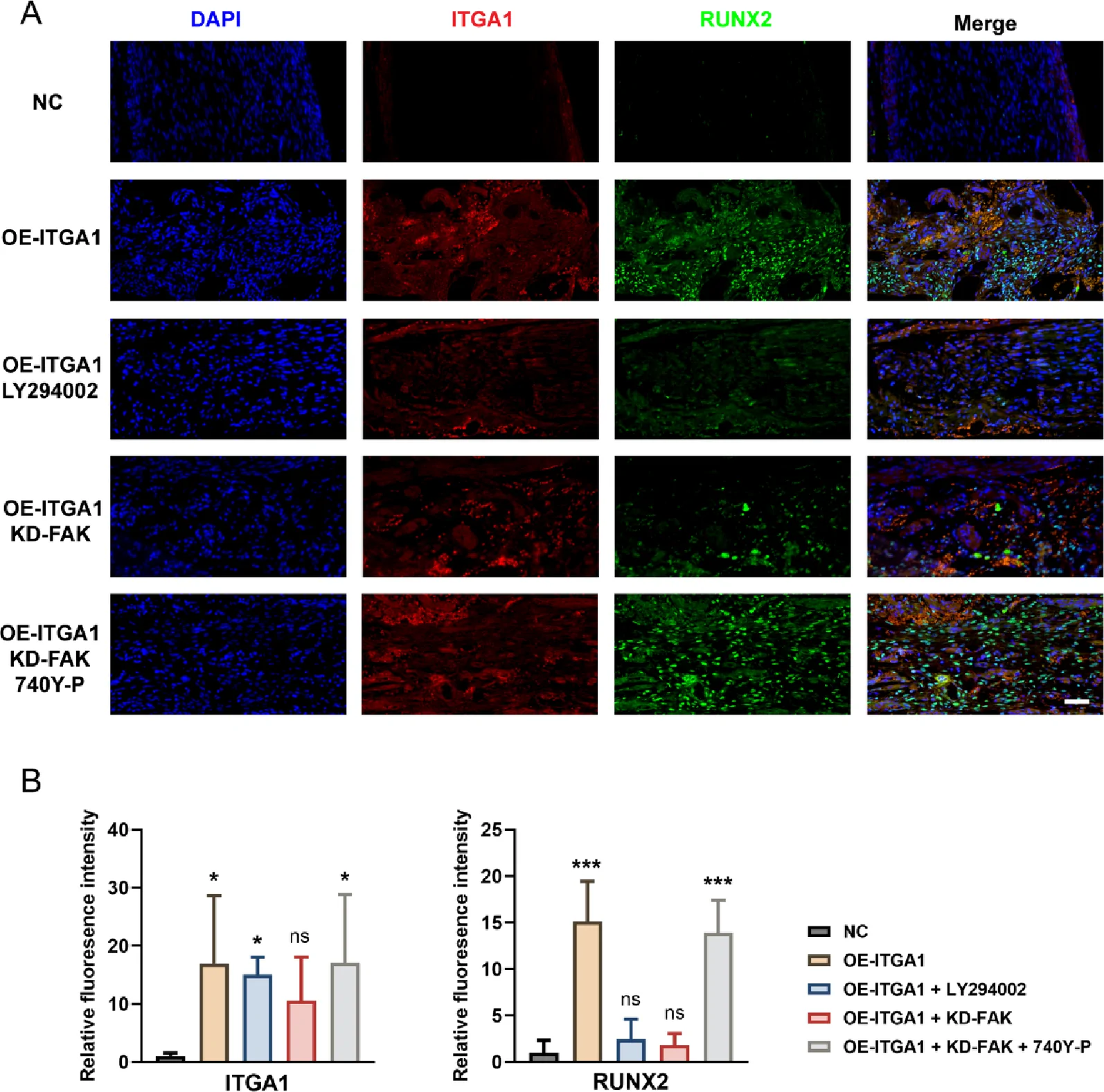
Immunofluorescence verification of ITGA1 enhancing osteogenesis of hPDLSCs. **A** Immunofluorescence staining of ITGA1 and RUNX2 expression in the mandibular bone defect area. ITGA1 (red), RUNX2 (green), and DAPI (blue). Scale bar = 50 μm. **B** Semi-quantitative analysis of immunofluorescence staining results performed using ImageJ (*n* = 6). Data are presented as mean ± SD. Statistical significance: *P < 0.05, **P < 0.01, ***P < 0.001

To evaluate the in vivo osteogenic potential of differentially treated hPDLSCs, the cells were delivered via a hydrogel into mandibular bone defects in rats (Fig. 7A). The results demonstrated that compared with the NC, OE-ITGA1 + LY294002, and OE-ITGA1 + KD-FAK hPDLSCs, the defects implanted with OE-ITGA1 or OE-ITGA1 + KD-FAK + 740 Y-P hPDLSCs markedly enhanced bone healing (Fig. 7B). Micro-CT analysis revealed significant increases in the BV/TV, whereas no significant difference in the Tb.Th, Tb.N, or Tb.Sp in these two groups (Fig. 7C). Histological examination further revealed abundant newly formed, collagen-rich bone matrix within the defect areas (Fig. 7D–E), along with stronger RUNX2 immunofluorescence signals in the newly regenerated tissues (Fig. 8A–B). Together, these results indicate that the ITGA1/FAK/PI3K–Akt signalling axis facilitates osteogenic differentiation of hPDLSCs and promotes bone regeneration in vivo.

## Discussion

Integrins serve as pivotal hubs for the transduction of mechanical and biochemical signals across the cell membrane and play a critical role in directing the differentiation of MSCs [32, 33]. However, the specific functions of individual integrin family members in osteogenesis remain incompletely understood. Lee et al. reported that integrin α3 and αV expression increased during osteogenic differentiation of human MSCs, concomitant with osteogenic marker upregulation. Moreover, compared with human MSCs with low integrin αV expression, those with high expression showed greater osteogenic potential [34]. Peng et al. reported that inhibiting integrin αVβ3 in human fibroblasts led to decreased ALP, RUNX2, and nuclear YAP expression, consequently suppressing osteogenic differentiation [35]. Liang et al. confirmed that integrin α10 regulates the adhesion, migration, and osteogenic differentiation of bone marrow-derived MSCs (BMSCs) from the alveolar bone of patients with type 2 diabetes after dental implantation surgery, suggesting that integrin α10 is a potential target for modulating BMSC function and osseointegration in these patients [36]. ITGA1, which heterodimerizes with the β1 subunit to form a receptor for collagens and laminin in the ECM [37], has been primarily studied in the context of cancer, where it is associated with cell adhesion, proliferation, and invasion [25, 38, 39]. In the present study, using adenovirus-mediated overexpression combined with loss-of-function approaches, we observed that ITGA1 positively regulates osteogenic differentiation in hPDLSCs. Additionally, an empty adenoviral vector was used as the negative control to minimize nonspecific effects associated with adenoviral transduction. These findings extend previous observations of integrin-mediated osteogenesis to hPDLSCs and provide further insight into the potential role of ITGA1 in this process.

To further explore the mechanism through which ITGA1 promotes osteogenic differentiation in hPDLSCs, DIA proteomics was performed. GO analysis revealed enrichment in “protein binding,” while KEGG analysis indicated enrichment in several signalling pathways associated with osteogenesis, including the PI3K–Akt pathway, suggesting that OE-ITGA1 may exert its effects by modulating protein interaction networks and intracellular signalling. In addition, to assess the specificity of ITGA1-mediated effects, ITGA5, which was upregulated in the proteomic analysis, was selected for functional validation. KD-ITGA5 did not abolish the pro-osteogenic effect of ITGA1, suggesting that this effect is not dependent on ITGA5. Based on these findings, PPI analysis was conducted, which highlighted focal adhesion kinase (FAK) as a potential key node. Subsequent IP–MS and Co-IP analyses supported the association between ITGA1 and FAK. FAK is a cytoplasmic nonreceptor protein tyrosine kinase and a central mediator of integrin-dependent signalling. It regulates multiple downstream pathways, including PI3K–Akt, Wnt, and MAPK, thereby influencing diverse cellular processes [40–42]. Previous studies have demonstrated that FAK phosphorylation is closely associated with osteogenic differentiation in various MSC populations. For instance, Salasznyk et al. demonstrated that FAK inhibition blocked osterix transcriptional activity and the osteogenic differentiation of human MSCs [43]. Zheng et al. reported that bone morphogenetic protein 9 promotes FAK phosphorylation and enhances osteogenic differentiation of synovial MSCs [44]. Chen et al. showed that matrix stiffness regulates integrin α2β1-mediated activation of FAK, thereby promoting osteogenesis [45]. Li et al. demonstrated that pentavalent vanadium ions regulate osteogenic differentiation of BMSCs through activation of the FAK/MAPK pathway [46]. Consistent with these studies, our results suggest that ITGA1 may promote osteogenic differentiation in hPDLSCs, at least in part, through activation of the FAK/PI3K–Akt signalling axis. This effect was attenuated upon silencing of FAK or inhibition of PI3K–Akt, whereas activation of PI3K–Akt partially restored osteogenic capacity under FAK-deficient conditions. Notably, although total FAK levels showed a decreasing trend upon OE-ITGA1, this may reflect increased protein turnover following activation, as indicated by enhanced phosphorylation. Consistently, treatment with the proteasome inhibitor MG132 partially restored FAK protein levels (Figure S1D–E), suggesting that proteasome-mediated degradation contributes to the dynamic regulation of FAK during this process.

Building on these cellular findings, we further evaluated the in vivo relevance of this mechanism using a rat mandibular bone defect model. The results showed that enhanced bone regeneration was observed following transplantation of OE-ITGA1 hPDLSCs, whereas inhibition of FAK or PI3K–Akt attenuated this effect. Partial restoration of bone formation was achieved upon reactivation of PI3K–Akt signalling. Although BV/TV was significantly increased, no significant differences were observed in Tb.N, Tb.Th, or Tb.Sp between groups as determined by micro-CT. This finding suggests that OE-ITGA1 may primarily enhance bone volume formation rather than markedly altering trabecular microarchitecture within the evaluated time frame. PI3K, as a key downstream effector of FAK, plays an essential role in intracellular signalling [47, 48]. Upon activation, PI3K catalyzes the conversion of PIP2 to PIP3, which recruits Akt and PDK1, leading to Akt phosphorylation and activation. Activated Akt subsequently regulates downstream effectors such as GSK-3β and mTOR, thereby influencing cell fate decisions [49–51]. Numerous studies have demonstrated that the PI3K–Akt pathway plays a central role in osteogenic differentiation of MSCs. In a previous study by our group, suppression of PI3K–Akt/β-catenin signalling was associated with reduced osteogenic capacity in embryonic MSCs from p75NTR knockout mice, whereas activation of this pathway promoted osteogenesis [52]. Similarly, Han et al. reported that protein crotonylation enhances osteogenic differentiation in PDLSCs via the PI3K–Akt pathway [53], and Zhao et al. demonstrated that exosomes derived from human umbilical cord MSCs promote osteogenesis of hBMSCs through activation of PI3K–Akt signalling [54].

**Fig. 9:**
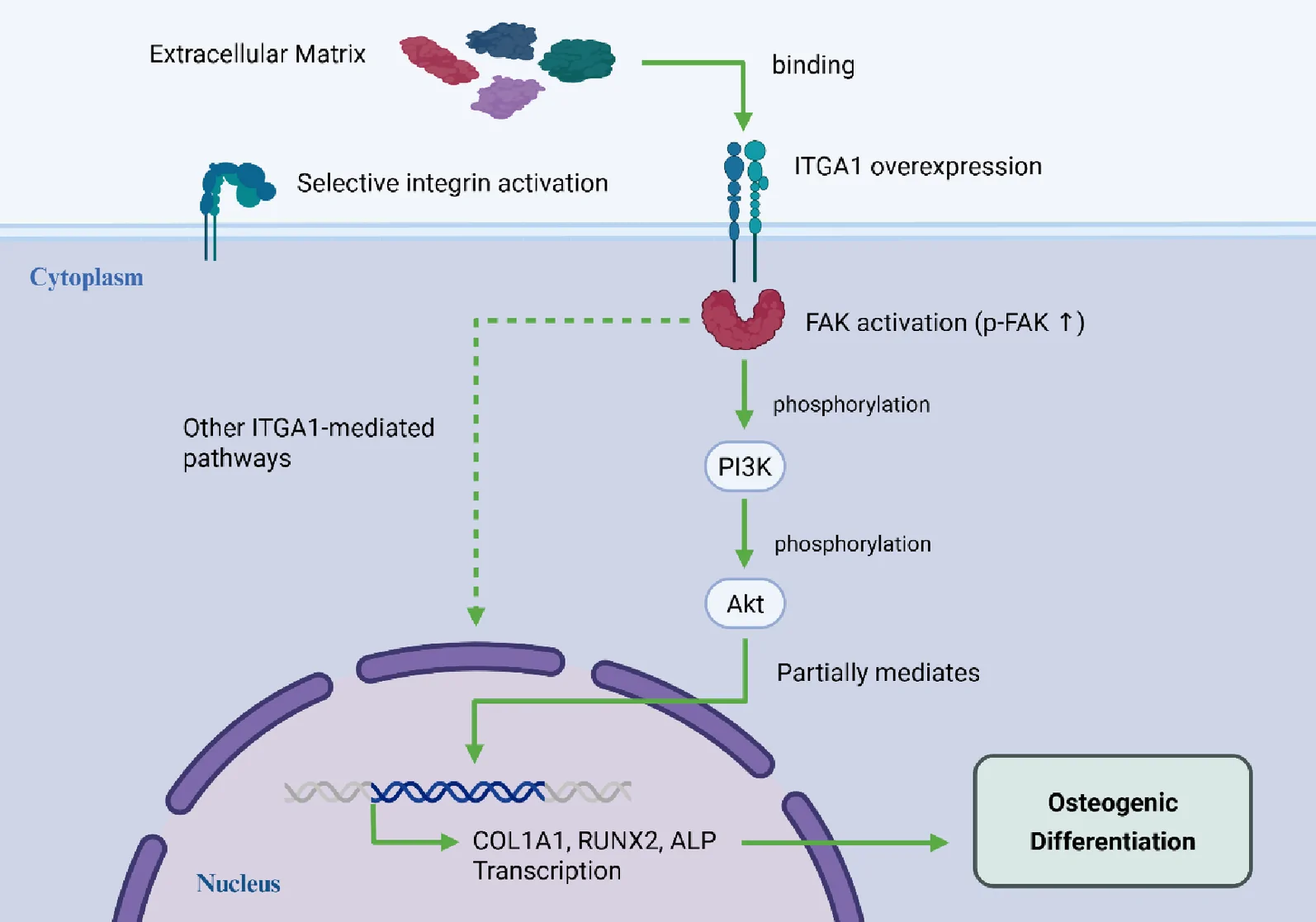
Mechanism of ITGA1-driven osteogenic differentiation in hPDLSCs. The schematic illustrates that ITGA1 promotes the osteogenic differentiation of hPDLSCs by interacting with FAK and activating the downstream PI3K–Akt signalling pathway. ITGA1-mediated signalling partially contributes to osteogenic gene transcription, while additional pathways may also be involved. The figure was created with BioRender.com

Our findings suggest that modulation of ITGA1 may enhance hPDLSC-mediated bone regeneration through the FAK/PI3K–Akt axis. The overall mechanism is illustrated in Fig. 9. This study, based on both in vitro and in vivo evidence, provides a mechanistic framework for understanding the role of ITGA1 in hPDLSC osteogenesis. However, several limitations should be acknowledged. First, adenovirus-mediated overexpression may not fully recapitulate physiological regulation of ITGA1. Second, although proteomic analyses were performed, additional ITGA1-associated pathways and interacting partners remain to be explored. Third, ITGA1 has been implicated in tumour progression in certain contexts, and the potential oncological risks associated with its modulation warrant further investigation. Fourth, donor variability of hPDLSCs may influence experimental outcomes, although consistent trends were observed across independent experiments. Additionally, extrapolation of in vitro findings to the in vivo rat mandibular defect model should be interpreted with caution due to microenvironmental complexity, and longer-term studies beyond the 4-week time point are needed to determine the persistence of these effects. Future studies should further investigate the structural basis of the ITGA1–FAK interaction and its regulation under different microenvironmental conditions, such as inflammation. Moreover, combining OE-ITGA1 hPDLSCs with biomimetic scaffolds or developing responsive delivery systems targeting the PI3K–Akt pathway may further improve regenerative outcomes.

## Conclusion

This study demonstrates that ITGA1 promotes osteogenic differentiation of hPDLSCs and is associated with activation of the FAK/PI3K–Akt signalling pathway. These findings provide additional insight into integrin-mediated regulation of osteogenesis and may offer a potential basis for the development of targeted strategies in bone tissue engineering.

## Supplementary Information

Below is the link to the electronic supplementary material.

Supplementary Material 1. Table S1 ITGA1 primer sequences.

Supplementary Material 2. Table S2 Primer sequences used in the qRT-PCR.

Supplementary Material 3. Table S3 Primer sequences for siRNA.

Supplementary Material 4. Table S4 Complete dataset of protein quantification and differential analysis from DIA-based proteomics.

Supplementary Material 5. Complete Dataset of IgG from IP–MS.

Supplementary Material 6. Complete Dataset of NC from IP–MS.

Supplementary Material 7. Complete Dataset of OE-ITGA1 from IP–MS.

Supplementary Material 8. Reagent list.

Supplementary Material 9. DIA - Materials and Methods.

Supplementary Material 10. ARRIVE checklist.

Supplementary Material 11. Fig. S1 Optimization and validation experiments related to OE-ITGA1, KD-FAK, signalling pathways regulation, and animal model establishment.

Supplementary Material 12. Fig. S2–S6 Original western blot images.

## Data Availability

The datasets used and/or analysed during the current study are available from the corresponding author on reasonable request.

## Abbreviations

hPDLSCs: Human periodontal ligament stem cells
MSC: Mesenchymal stem cell
ECM: Extracellular matrix
ITGA1: Integrin α1
p75NTR^+^: p75 neurotrophin receptor-positive
KD: Knocked down
OE: Overexpression
DEPs: Differentially expressed proteins
FBS: Foetal bovine serum
ALP: Alkaline Phosphatase
MOI: Multiplicity of infection
qRT-PCR: Quantitative real-time polymerase chain reaction
FAK: Focal adhesion kinase
p-FAK: Phospho-FAK
p-PI3K: Phospho-PI3K
p-Akt: Phospho-Akt
DIA: Data-independent acquisition
FC: Fold change
PPI: Protein–protein interaction
Co-IP: Coimmunoprecipitation
IP–MS: Immunoprecipitation–mass spectrometry
siRNA: Small interfering RNA
SD rat: Sprague–Dawley rat
BV/TV: Bone volume/total volume
Tb.N: Trabecular number
Tb.Th: trabecular thickness
Tb.Sp: Trabecular separation
H༆E: Haematoxylin and eosin
SD: Standard deviation
one-way ANOVA: One-way analysis of variance
WT: Wild-type
GO: Gene Ontology
KEGG: Kyoto Encyclopedia of Genes and Genomes
BMSCs: Bone marrow-derived MSCs
