# Supplementary material for: ITGA1 promotes osteogenic differentiation of human periodontal ligament stem cells via FAK-mediated PI3K–Akt activation
Source: Stem Cell Res Ther. 2026 Jul 3;17:334. doi: 10.1186/s13287-026-05139-6 (PMC13613795; doi:10.1186/s13287-026-05139-6)
Supplement: Supplementary file 2 [file 13287_2026_5139_MOESM6_ESM.pdf]

DIA - Materials and Methods

1.1 Reagents

The following samples were used for the experiment: iRTkit and Bradford protein quantification kit (both purchased from Biognosys), DTT (purchased from Sigma/D9163-25G), IAM (purchased from Sigma/I6125-25G), SDS (purchased from China National Pharmaceutical), urea (purchased from National Drug/10023218), mass spectrometry grade trypsin (purchased from Promega/V5280), ammonium bicarbonate (purchased from Sigma/5330050050), LC-MS grade ultrapure water (purchased from ThermoFisherChemical/W6-4), TEAB buffer (purchased from Sigma/T7408-500ML), LC-MS grade acetonitrile (purchased from ThermoFisherChemical/A955-4), LC-MS grade formic acid (purchased from Thermo FisherScientific/A117-50), acetone (purchased from Beijing Chemical Factory/11241203810051), ammonia (purchased from Sigma/T7408-500ML), and water (purchased from Sigma/T7408-221228-500ML-A). Additionally, the experiment utilized the HighSelectTop14AbundantProteinDepletionMiniSpinColumns kit (purchased from Thermo Fisher/A36370, NP) and trifluoroacetic acid (TFA, purchased from Sigma/T6508-100ML).

1.2 Instrumentation

The liquid phase Vanquish™ NeoUHPLC (purchased from ThermoFisher), mass spectrometer OrbitrapAstral mass spectrometer (purchased from ThermoFisher), cryogenic centrifuge (purchased from Scilogex/D3024R), freeze dryer (purchased from Labogene/ScanSpeed40), electrophoresis machine and tank (both purchased from Bio-Rad), electronic balance (purchased from Sartorius/BSA124S), vortex mixer (purchased from Photosynthesis/HY-6B), enzyme labeling instrument (purchased from Thermo/MultiskanFC), ice maker (purchased from Xueke), tissue milling instrument (purchased from Shanghai Jingshun/24 holes), and ultrasonic cell crusher (purchased from Ningbo Xinzhi/JY92-11N) were used in the experiment.

2. Total Protein Extraction

2.1 Animal tissue [1-3]

Sample was ground individually in liquid nitrogen and lysed with SDT (containing 100 mM Nacl) and 1/100 volume of DTT, followed by 5 min of ultrasonication on ice. After incubation 8-15min at 95℃ and ice-bath for 2min, the lysate was centrifuged at 12000 *g* for 15 min at 4℃ and the supernatant was subsequently alkylated with sufficient IAM for 1 h at room temperature in the dark.

Then samples were completely mixed with 4 times volume of precooled acetone by vortexing and incubated at -20°C for at least 2 h. Samples were then centrifuged at 12000 *g* for 15 min at 4°C and the precipitation was collected. After washing with 1mL cold acetone, the pellet was dissolved completely by Dissolution Buffer (DB buffer).

2.2 Cell [1-3]

The sample was transferred it to a 1.5ml centrifuge tube and lysed with DB lysis buffer (8 M Urea, 100 mM TEAB, pH 8.5), followed by 5 min of ultrasonication on ice. The lysate was centrifuged at 12000 *g* for 15 min at 4°C and the supernatant was added with 1M DTT to react for 1 h at 56°C, and subsequently alkylated with sufficient iodoacetamide for 1 h at room temperature in the dark followed by ice-bath for 2min.

2.3 Cell culture supernatant [1-3]

Some sample was taken into an ultrafiltration tube and centrifuged at 14000 *g* for 15 min. After discarding the permeate, the ultra-filtered sample was put into a 1.5ml centrifuge tube and balanced with DB buffer (8 M Urea, 100 mM TEAB, pH 8.5). Took the supernatant and added 1 M DTT to react for 1 h at 56°C, and subsequently alkylated with sufficient IAM for 1 h at room temperature in the dark.

2.4 Plant tissue [4-8]

Sample was ground individually in liquid nitrogen and lysed with SDT lysis buffer (containing 100 mM Nacl) and 1/100 volume of DTT, followed by 5 min of ultrasonication on ice. After reacting at 95°C for 8-15 min and ice-bath for 2min, the lysate was centrifuged at 12000 *g* for 15 min at 4°C. And the supernatant was taken and added with sufficient IAM to react for 1 h at room temperature in the dark. Then samples were completely mixed with 4 times volume of precooled acetone by vortexing and incubated at -20°C for at least 2 h. Samples were then centrifuged at 12000 *g* for 15 min at 4°C and the precipitation was collected. After washing with 1mL cold acetone, the pellet was completely dissolved by Dissolution Buffer (DB buffer).

2.5 Bacteria [4-8]

The sample was grounded to powder at low temperature and transferred immediately to the liquid nitrogen-precolded centrifuge tube and lysed with SDT lysis buffer and 1/100 volume of DTT, followed by 5 min of ultrasonication on ice. After incubation for 8-15min at 95°C and ice-bath for 2min, the lysate was centrifuged at 12000 *g* for 15 min at 4°C, and subsequently the supernatant was added with sufficient IAM to react for 1 h in the dark. Then samples were completely mixed

with 4 times volume of precooled acetone by vortexing and incubated at -20°C for at least 2 h. Samples were then centrifuged at 12000 *g* for 15 min at 4°C and the precipitation was collected. After washing with 1mL cold acetone, the pellet was completely dissolved by Dissolution Buffer (DB buffer).

2.6 Blood

2.6.1 Remove high-abundance proteins [9-10]

To prepare the sample, invert Spintip's beads and remove the top cover. Then, add the required amount of blood (serum, plasma) sample to the resin slurry supernatant and mix thoroughly. Allow the reaction to mix at room temperature for 10 minutes. Afterward, discard the bottom plug and place Spintip on top of a 1.5ml centrifuge tube. Centrifuge at 1000g for 2 minutes and discard the top resin tube. Next, add an appropriate amount of penetration solution and centrifuge at 13,000g for 10 minutes. The resulting supernatant will be the extracted protein. Finally, add an appropriate amount of TEAB, TCEP, and CAA to the protein solution and mix thoroughly. Place the solution in a 95°C metal bath for 10 minutes.

2.6.2 NP(Nanoparticle) remove high-abundance proteins（ HD Blood- Plus）

To enrich plasma or serum protein, aspirate 500μg of nanomagnetic material magnetic beads (50μl volume) and perform magnetic separation to remove the supernatant. Wash the beads once with a certain amount of wash buffer. Then, add an equal volume of wash buffer and plasma, and incubate at 37℃ with shaking at 1500rpm for 1 hour. Perform magnetic separation to remove the supernatant, and wash the magnetic beads three times with three times the volume of wash buffer. Shake for 5 minutes during each wash. Repeat this process three times. The top of the magnetic beads will contain the enriched plasma or serum protein.

2.6.3 Don' t remove high-abundance proteins

The sample was transferred to a 1.5ml centrifuge tube and lysed with some DB lysis buffer (8 M Urea, 100 mM TEAB, pH 8.5). The lysate was centrifuged at 12000 *g* for 15 min at 4°C and the supernatant was reduced with 1M DTT for 1 h at 56°C, and subsequently alkylated with sufficient IAM for 1 h at room temperature in the dark followed by ice-bath for 2min.

2.7 FFPE [11]

Samples are dewaxed using the FFPE Sample Dewaxing Kit. After removing the PBS solution, an appropriate amount of protein lysate (4% SDS, 100 mM Tris) was added and incubated at 95°C for 10 minutes at room temperature, mixed by shaking, and sonicated in an ice-water bath for 5

minutes. The samples were de-crosslinked with a refractive index at 95℃ for 60 min, then reduced by adding appropriate amount of TCEP, and alkylated in CAA at 95℃ for 5min. The samples were sequentially centrifuged at 12000 *g* at 4℃ for 15 min. Collecting the supernatant and adding 4 times volume of pre-cooling acetone at -20℃, and precipitated it at -20℃ for at least 4h. Centrifuging at 12000 *g* for 15 min at 4℃. Collecting the precipitate and air drying. Appropriate amount of protein solution (8 M urea, 100 mM TEAB, pH = 8.5) was added to dissolve the protein pellet.

3. Protein Quality Test

BSA standard protein solution was prepared according to the instructions of Bradford protein quantitative kit, with gradient concentration ranged from 0 to 0.5 g/L. BSA standard protein solutions and sample solutions with different dilution multiples were added into 96-well plate to fill up the volume to 20 µL, respectively. Each gradient was repeated three times. The plate was added 180 µL G250 dye solution quickly and placed at room temperature for 5 minutes, the absorbance at 595 nm was detected. The standard curve was drawn with the absorbance of standard protein solution and the protein concentration of the sample was calculated. 20 µg of the protein sample was loaded to 12% SDS-PAGE gel electrophoresis, wherein the concentrated gel was performed at 80 V for 20 min, and the separation gel was performed at 120 V for 90 min. After completing the electrophoresis, the samples were stained using Caulmers Brilliant Blue Staining Riso Reagent and decolorized until the bands became clearly visible.

4. Trypsin treatment [12]

Each protein sample was taken and the volume was made up to 100 µL with DB lysis buffer (8 M Urea, 100 mM TEAB, pH 8.5), trypsin and 100 mM TEAB buffer were added, sample was mixed and digested at 37 °C for 4 h. Then trypsin were added digested overnight. Formic acid was mixed with digested sample, adjusted pH under 3, and centrifuged at 12000 *g* for 5 min at room temperature. The supernatant was slowly loaded to the C18 desalting column, washed with washing buffer (0.1% formic acid, 3% acetonitrile) 3 times, then added elution buffer (0.1% formic acid, 70% acetonitrile). The eluents of each sample were collected and lyophilized.

Table 1 Liquid chromatography elution gradient table

Time	flow rate (nL/min)	mobile phase A (%)	mobile phase B（%）
0	2.5	96	4

0.2	1.3	96	4
0.3	0.8	92	8
0.5	0.8	92	8
14.2	0.8	77.5	22.5
21.1	0.8	65	35
21.5	2.5	45	55
21.5		Column Wash	
21.9	2.5	1	99
22.6	2.5	1	99
22.6		Stop Run	

5. LC-MS/MS Analysis-DIA mode

5.1 Vanquish™ Neo UHPLC -Astral LC/MS DIA method

Prepare mobile phase A (100% water, 0.1% formic acid) and B (80% acetonitrile, 0.1% formic acid). The lyophilized powder was dissolved using 10μ LA solution, centrifuged at 14,000g for 20 min at 4°C, and 200 ng of the supernatant sample was injected into the sample for liquid-quality detection. A Vanquish Neo upgraded UHPLC system was used with a C18 pre-column of 174500 (5 mm×300 μm ,5 μm, thermo) heated at 50°C in a column oven, and a C18 analytical column of ES906 (PepMap TM Neo UHPLC 150μm x 15 cm, 2 μm, thermo), and the elution conditions of the liquid chromatography were as shown in Table 1, a Thermo orbitrap astral mass spectrometer mass spectrometer was used, an Easy-spray (ESI) ion source was used, the ion spray voltage was set to 2.0 kV, the ion transfer tube temperature was set to 290°C, and the mass spectrum was in a data-independent acquisition mode, with a full first-stage mass spectrometry scanning range of m/z 380-980. The primary MS resolution was set to 240000 (200m/z), AGC was set to 500%, the parent ion window size was set to 2-Th, the number of DIA windows was 300, the NCE was set to 25%, the secondary m/z acquisition range was from 150 to 2000, the sub-ion resolution Astral was set to 80000, and the maximal injection time was 3ms. Into mass spectrometry detection raw data (.raw).

6. Data analysis

6.1 The identification and quantitation of protein

The raw files were searched and analyzed using the DIA-NN library search software, according to the *** protein database. The library search parameters were set as follows: a mass tolerance of

10 ppm for precursor ions and 0.02 Da for fragment ions. Cysteine was modified by alkylation, methionine was oxidatively modified, and N-terminal modifications included acetylation, loss of methionine, and loss of methionine + acetylation. Two missed cleavage site was allowed at most.

To improve the quality of the analytical results, the DIA-NN software further filtered the search results by retaining only credible Peptide Spectrum Matches (PSMs) with a confidence level of 99% or higher. Only credible spectral peptides and proteins were retained, and FDR validation was performed to remove peptides and proteins with an FDR greater than 1%.A protein with a fold change (FC) greater than or less than a certain value (FC) was defined as a differentially expressed protein (DEP).

Note: For HD Blood-Plus (Astral platform), the DIA-NN software (DirectDIA) was used for raw data deconvolution and species library searching [13]. Retention time correction was performed using iRT added to the samples, and the precursor ion Qvaluecutoff value was set to 0.01. Protein quantification results were statistically analyzed using the T-test, and differentially expressed proteins were defined as those with significant quantitative differences between the experimental and control groups ($p<0.05$,| log2FC|>*(FC) or FC<*(fold change, FC)).。

6.2 The functional analysis of protein and DEP

Gene Ontology (GO) and InterPro (IPR) functional analysis were conducted using the interproscan program against the non-redundant protein database (including Pfam, PRINTS, ProDom, SMART, ProSite, PANTHER) [15], and the databases of COG (Clusters of Orthologous Groups) and KEGG (Kyoto Encyclopedia of Genes and Genomes) were used to analyze the protein family and pathway. DEPs were used for Volcanic map analysis, cluster heat map analysis and enrichment analysis of GO, IPR and KEGG [16].

Reference:

[1] Kachuk, Carolyn, Stephen, et al. Comparison of sodium dodecyl sulfate depletion techniques for proteome analysis by mass spectrometry[J]. Journal of chromatography, A: Including electrophoresis and other separation methods, 2015.

[2] Wiśniewski, Jacek R, Zoμgman, et al. Universal sample preparation method for proteome analysis[J]. Nature Methods, 2009.

[3] Satpathy S, Mannan R, Ana R I. Proteogenomic Characterization Reveals Therapeutic Vulnerabilities in Lung Adenocarcinoma[J]. Cell, 2020, 182(1).

[4] Kachuk, Carolyn, Stephen, et al. Comparison of sodium dodecyl sulfate depletion techniques

for proteome analysis by mass spectrometry[J]. Journal of chromatography, A: Including electrophoresis and other separation methods, 2015.

[5] Wu X, Xiong E, Wang W, et al. Universal sample preparation method integrating trichloroacetic acid/acetone precipitation with phenol extraction for crop proteomic analysis[J]. Nature Protocols, 2014, 9(2):362.

[6] Liangjie, Niu, Hang, et al. Modified TCA/acetone precipitation of plant proteins for proteomic analysis[J]. PLoS ONE, 2018.

[7] Winiewski J R, Zoµgman A, Nagaraj N, et al. Universal sample preparation method for proteome analysis[J]. Nature Methods, 2009, 6(5):359-362.

[8] Marx H, Minogue C E, Jayaraman D, et al. A proteomic atlas of the legume Medicago truncatula and its nitrogen-fixing endosymbiont Sinorhizobium meliloti[J]. Nature Biotechnology, 2016.

[9] Silva‐Costa L C, Garcia‐Rosa S, Smith B J, et al. Blood plasma high abundant protein depletion unintentionally carries over 100 proteins[J]. SEPARATION SCIENCE PLUS, Wiley, 2019, 2(12): 449–456.

[10] Sanchez J G, Okreglicka K, Chandrasekaran V, et al. The tripartite motif coiled-coil is an elongated antiparallel hairpin dimer[J]. Proceedings of the National Academy of Sciences, Proceedings of the National Academy of Sciences, 2014, 111(7): 2494–2499.

[11] Buczak K, Kirkpatrick J M, Truckenmueller F, et al. Spatially resolved analysis of FFPE tissue proteomes by quantitative mass spectrometry[J]. Nature Protocols, 2020:1-24.

[12] Zhang H, Liu T, Zhang Z, et al. Integrated Proteogenomic Characterization of Human High-Grade Serous Ovarian Cancer[J]. Cell, 2016.

[13] Demichev, V., Messner, C.B., Vernardis, S.I. *et al.*DIA-NN: neural networks and interference correction enable deep proteome coverage in high throughput. *Nat Methods* **17,** 41–44 (2020).

[14] Jones P, Binns D, Chang H Y, et al. InterProScan 5: genome-scale protein function classification[J]. Bioinformatics, 2014, 30(9): 1236-1240.

[15] Huang D W, Sherman B T, Lempicki R A. Bioinformatics enrichment tools: paths toward the comprehensive functional analysis of large gene lists[J]. Nucleic Acids Research, 2009, 37(1): 1-13.

[16] Franceschini A, Szklarczyk D, Frankild S, et al. STRING V9.1: Protein-Protein Interaction Networks, with Increased Coverage and Integration[J]. Nucleic Acids Research, 2012, 41(D1).

Notice:

1> The description of this method is just for reference and cannot be used directly for the publication of the article. You need to write it according to the original intention；

2> The yellow mark in the description needs to be filled in according to the actual project situation of the project.

Novogene 诺禾致源
