# Supplementary material for: ITGA1 promotes osteogenic differentiation of human periodontal ligament stem cells via FAK-mediated PI3K–Akt activation
Source: Stem Cell Res Ther. 2026 Jul 3;17:334. doi: 10.1186/s13287-026-05139-6 (PMC13613795; doi:10.1186/s13287-026-05139-6)
Supplement: Supplementary file 3 [file 13287_2026_5139_MOESM10_ESM.docx]

**Table S3** Primer sequences for siRNA

| Name | Sequence (5’–3’) |
| --- | --- |
| hITGA1-123 | Sense: GCUGCUGCGUAUCAUUCAATT  Antisense: UUGAAUGAUACGCAGCAGCTT |
| hITGA5-527  hFAK-2828 | Sense: GCUACCUCUCCACAGAUAATT  Antisense: UUAUCUGUGGAGAGGUAGCTT  Sense: CCGAUUGGAAACCAACAUAUATT  Antisense: UAUAUGUUGGUUUCCAAUCGGTT |
| NC | Sense: UUCUCCGAACGUGUCACGUTT  Antisense: ACGUGACACGUUCGGAGAATT |
