# Supplementary material for: ITGA1 promotes osteogenic differentiation of human periodontal ligament stem cells via FAK-mediated PI3K–Akt activation
Source: Stem Cell Res Ther. 2026 Jul 3;17:334. doi: 10.1186/s13287-026-05139-6 (PMC13613795; doi:10.1186/s13287-026-05139-6)
Supplement: Supplementary file 4 [file 13287_2026_5139_MOESM12_ESM.docx]

**Supplementary Fig. S2**

**Supplementary Fig. S2** Full-length blot corresponding to Figure 3F. The cropped area used in the main figure is indicated by a black rectangle. (a) COL1A1 (b) GAPDH (c) ITGA1 (d) GAPDH (e) FAK (f) GAPDH.

**Supplementary Fig. S3**

**Supplementary Fig. S3** Full-length blot corresponding to Figure 4E. The cropped area used in the main figure is indicated by a black rectangle. (a) ITGA1 (b) GAPDH (c) ITGA5 (d) GAPDH (e) PI3K (f) p-PI3K (g) GAPDH (h) Akt (i) p-Akt (j) GAPDH (k) COL1A1 (l) ALP (m) GAPDH (n)RUNX2 (o) GAPDH

**Supplementary Fig. S4**

**Supplementary Fig. S4** Full-length blot corresponding to Figure 5C. The cropped area used in the main figure is indicated by a black rectangle. (a) Input-ITGA1 (b) Input-FAK (c) Input-GAPDH (d) IP:ITGA1-FAK (e) IP:ITGA1-ITGA1 (f) Input-ITGA1 (g) Input-FAK (h) Input-GAPDH (i) IP:FAK-ITGA1 (j) IP:FAK-FAK

**Supplementary Fig. S5**

**Supplementary Fig. S5** Full-length blot corresponding to Figure 6D. The cropped area used in the main figure is indicated by a black rectangle. (a) ITGA1 (b) GAPDH (c) FAK (d) GAPDH (e) p-FAK (f) GAPDH (g) PI3K (h) p-PI3K (i) GAPDH (j) AKT (k) p-AKT (l)GAPDH (m) COL1A1 (n) GAPDH (o) RUNX2 (p) ALP(q) GAPDH

**Supplementary Fig. S6**

**Supplementary Fig. S6** Full-length blot corresponding to Figure S1D. The cropped area used in the main figure is indicated by a black rectangle. (a) FAK (b) GAPDH
