# Supplementary material for: ITGA1 promotes osteogenic differentiation of human periodontal ligament stem cells via FAK-mediated PI3K–Akt activation
Source: Stem Cell Res Ther. 2026 Jul 3;17:334. doi: 10.1186/s13287-026-05139-6 (PMC13613795; doi:10.1186/s13287-026-05139-6)
Supplement: Supplementary file 5 [file 13287_2026_5139_MOESM7_ESM.docx]

**Fig. S1** Optimization and validation experiments related to OE-ITGA1, KD-FAK, signaling pathways regulation, and animal model establishment. (**A**) ALP staining of hPDLSCs transduced with OE-ITGA1 adenovirus at different MOI after 5 days of osteogenic induction. Scale bar = 100 μm. (**B**) Semi-quantitative analysis of ALP staining intensity measured using ImageJ (n = 3). (**C**) Relative mRNA expression levels of *ITGA1*, *COL1*, *RUNX2*, and *ALP* in hPDLSCs after 5 days of osteogenic induction, analyzed by qRT-PCR. *GAPDH* was used as an internal control (n = 3). (**D**) Western blot analysis of FAK protein levels following MG132 treatment (Full-length blots are presented in Supplementary Fig. S6). (**E**) Semi-quantitative analysis of the Western blot results using ImageJ (n = 3). (**F**) Relative *FAK* mRNA expression in hPDLSCs after transfection with FAK siRNA, determined by qRT-PCR and normalized to GAPDH (n = 3). (**G**–**H**) Semi-quantitative analysis of PI3K, p-PI3K, Akt, and p-Akt protein levels corresponding to Fig. 4F (G) and Fig. 6E (H), respectively (n = 3). (**I**) Surgical procedure for the rat mandibular bone defect model: creation of the defect, implantation of hPDLSCs mixed with hydrogel, and wound closure. Data are presented as mean ± SD. Statistical significance: **P* < 0.05, ***P* < 0.01, ****P* < 0.001
