# Supplementary material for: ITGA1 promotes osteogenic differentiation of human periodontal ligament stem cells via FAK-mediated PI3K–Akt activation
Source: Stem Cell Res Ther. 2026 Jul 3;17:334. doi: 10.1186/s13287-026-05139-6 (PMC13613795; doi:10.1186/s13287-026-05139-6)
Supplement: Supplementary file 6 [file 13287_2026_5139_MOESM8_ESM.docx]

**Table S1** ITGA1 primer sequences

| Name | Sequence (5’–3’) |
| --- | --- |
| Primer 1（＋） | AACCGTCAGATCTCTAGAGCCACCATGGCCCCTCGGCC |
| Primer 2（－）  Primer ID（＋）  Primer ID（－） | CAGAGGTTGATTATCGATTCATTTCTCCATTTTCTTTTTCAGTG  GAAGACACGATTTATGAAGCTGA  GAAATTTGTGATGCTATTGC |

Primer pair 1 (+, –): PCR amplification of the ITGA1 gene.

Primer pair ID (+, –): Colony PCR identification of positive transformants.
