# Supplementary material for: ITGA1 promotes osteogenic differentiation of human periodontal ligament stem cells via FAK-mediated PI3K–Akt activation
Source: Stem Cell Res Ther. 2026 Jul 3;17:334. doi: 10.1186/s13287-026-05139-6 (PMC13613795; doi:10.1186/s13287-026-05139-6)
Supplement: Supplementary file 7 [file 13287_2026_5139_MOESM9_ESM.docx]

**Table S2** Primer sequences for qRT-PCR

| Genes | Forward primer (5’–3’) | Reverse primer (5’–3’) |
| --- | --- | --- |
| *ITGA1*  *ITGA5*  *ALP*  *FAK*  *COL1A1*  *RUNX2*  *GAPDH* | AAGTGACCATGAATTTTGAGCC  GCTTCAACTTAGACGCGGAG  CTGGTACTCAGACAACGAGATG  AGTGGACCAGGAAATTGCTTTG  GAGGGCCAAGACGAAGACATC  GCGCATTCCTCATCCCAGTA  GTGGACCTGACCTGCCGTCTAG | CACATCAAAACACACTGTAGCA  GCACACTGACCCCGTCTG  GTCAATGTCCCTGATGTTATGC  GTGTTTTGGCCTTGACAGAATC  CAGATCACGTCATCGCACAAC  GGCTCAGGTAGGAGGGGTAA  GTGTCGCTGTTGAAGTCAGAGGAG |
